# Supplementary material for: A microengineered vascularized bleeding model that integrates the principal components of hemostasis
Source: Nat Commun. 2018 Feb 6;9:509. doi: 10.1038/s41467-018-02990-x (PMC5802762; doi:10.1038/s41467-018-02990-x)
Supplement: Supplementary file 1 — Supplementary Information [file 41467_2018_2990_MOESM1_ESM.pdf]

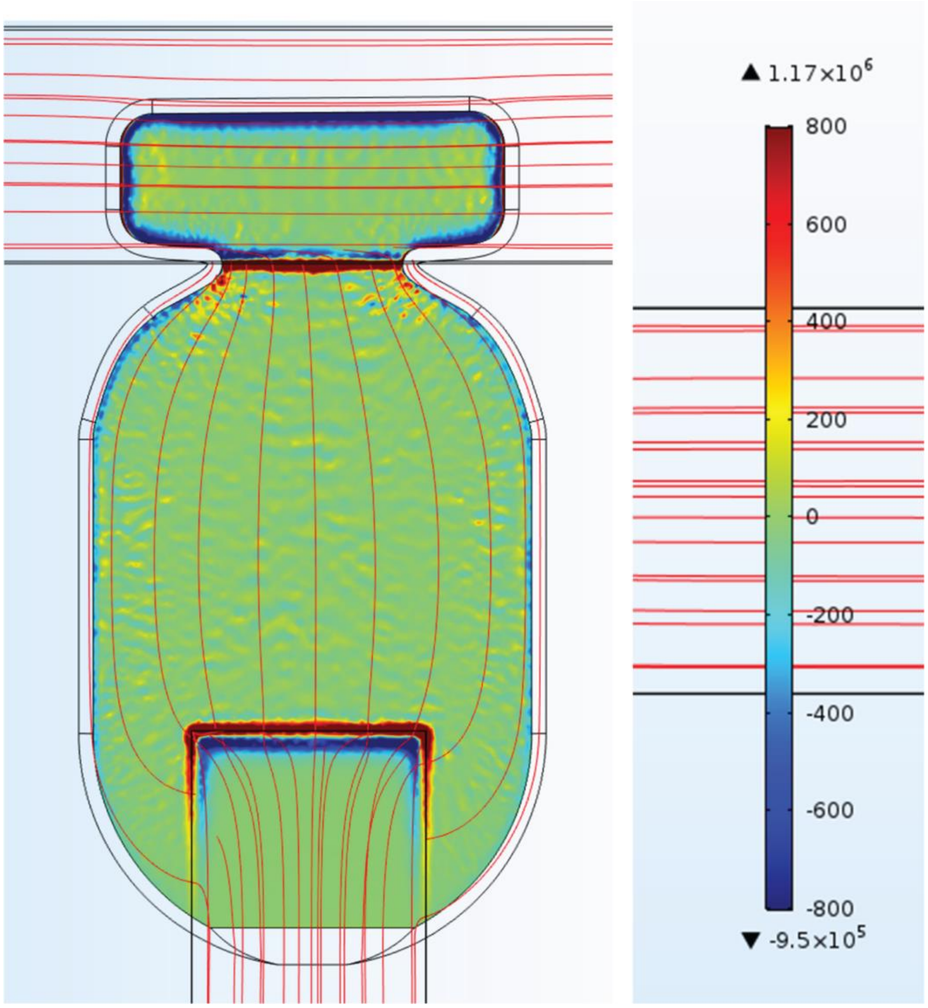

Supplementary Figure 1. COMSOL analysis of the shear gradient shows the highest shear occurs at the site of injury initiation. As expected, the shear gradient is highest where blood flows from the vascular channel into the wound channel. Red lines represent streamlines of fluid flow. Units for the color bar on the right are 1/mm·s.

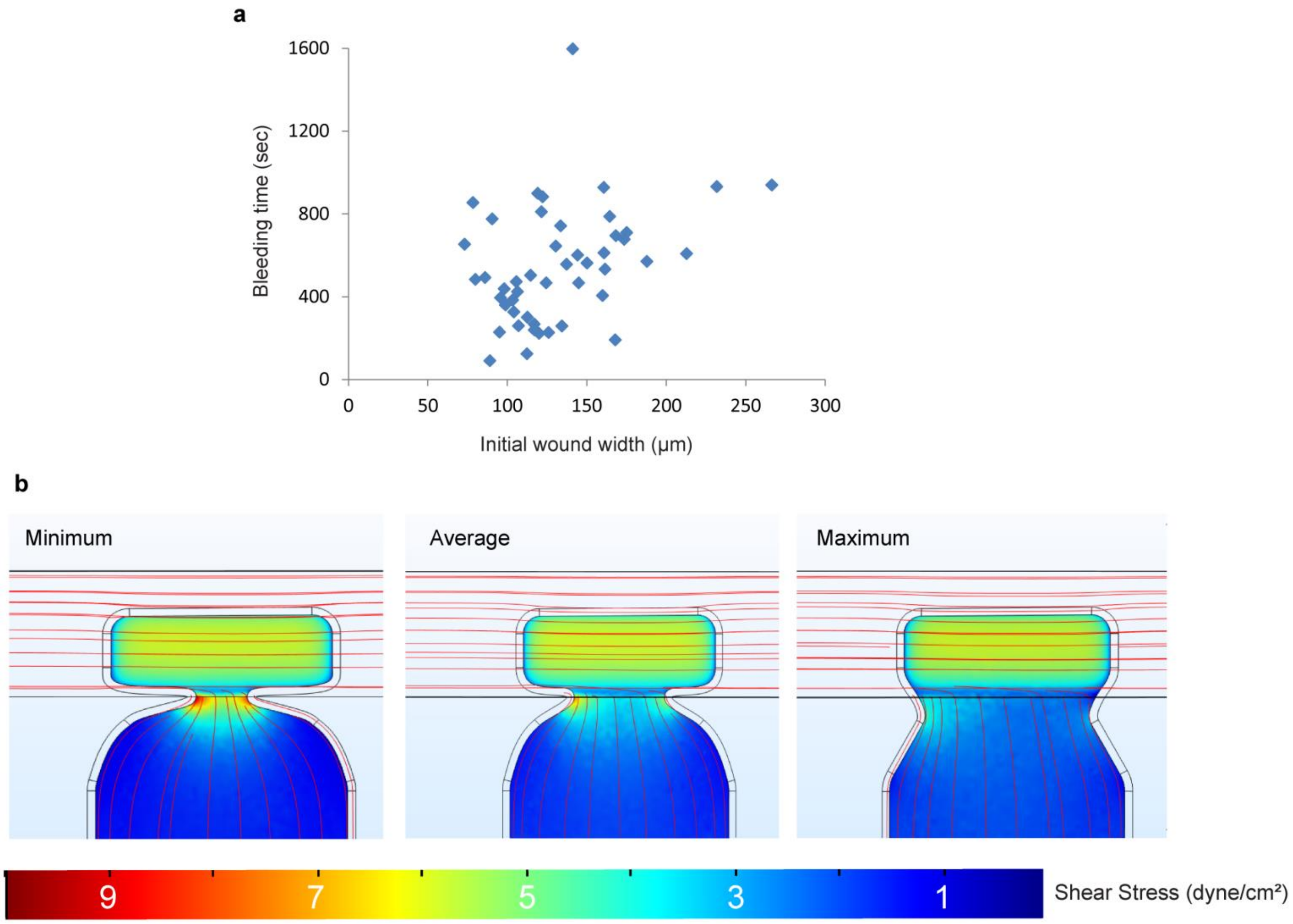

Supplementary Figure 2. The initial wound width in our mechanical injury bleeding model does not correlate with bleeding time. (a)The widths of the wound and the bleeding times from total 47 experiments with healthy whole blood were plotted. No correlation was observed between initial wound width and bleeding time (R^2= 0.088). (b) COMSOL simulation shows that shear stress profiles are all within the same order of magnitude over the operable range of wound widths (minimum 73.22, average 132.49, and maximum 231.86 μm).

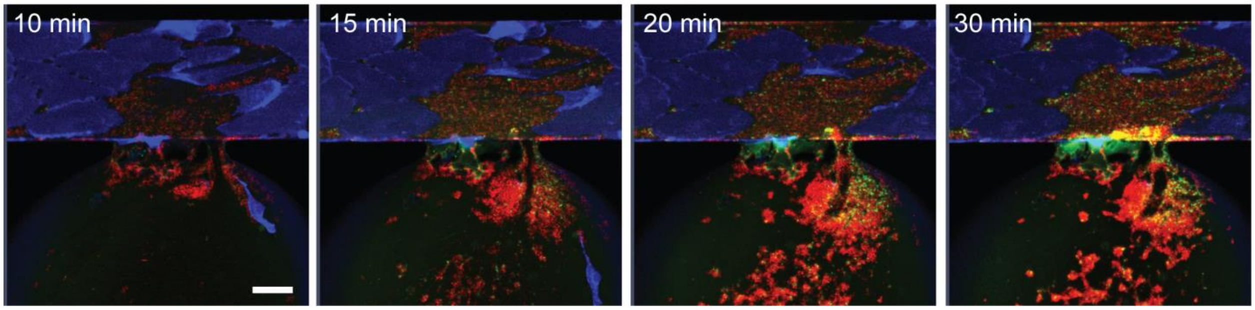

Supplementary Figure 3. Our mechanical injury bleeding model can be vascularlized by human aortic endothelial cells (HAECs) and show similar course of hemostasis. HAECs were seeded in the vascular channel and blood was perfused at the average aortic shear rate of 2500 s^{-1}, five times higher than that utilized in our studies using HUVECs (500 s^{-1}) and we observed platelet adhesion (red) and fibrin accumulation (green) in a similar manner. Scale bar = 50 µm.

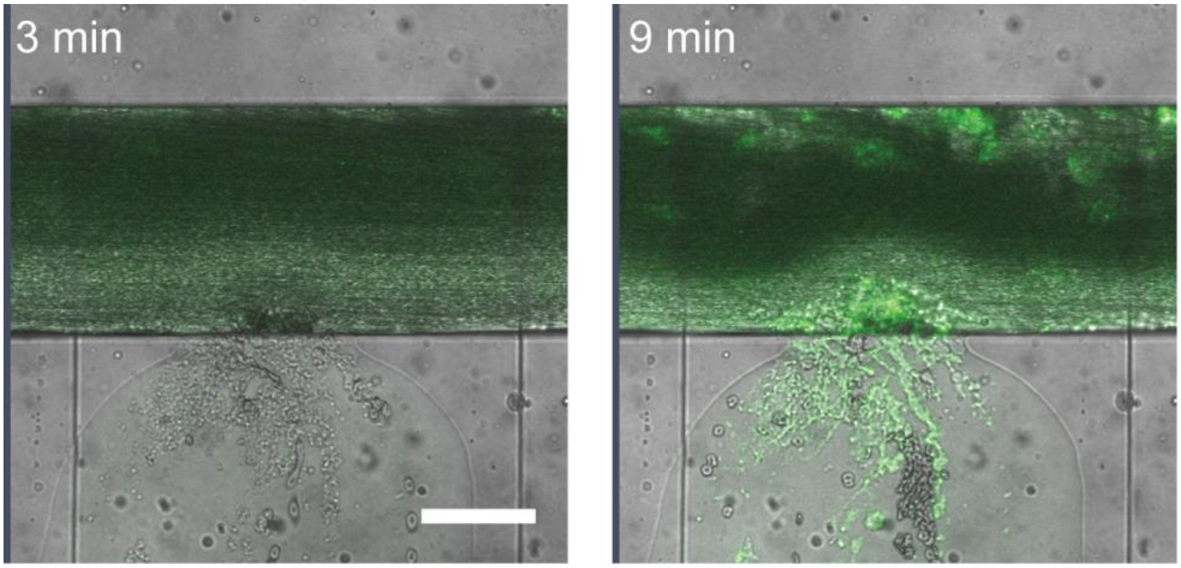

Supplementary Figure 4. P-selectin is exposed while hemostasis is achieved. While there are no P-selectin observed initially on platelets adhered at wound site (left), we observed P-selectin during hemostasis is achieved in later time point (green, right). Scale bar = 50 µm.

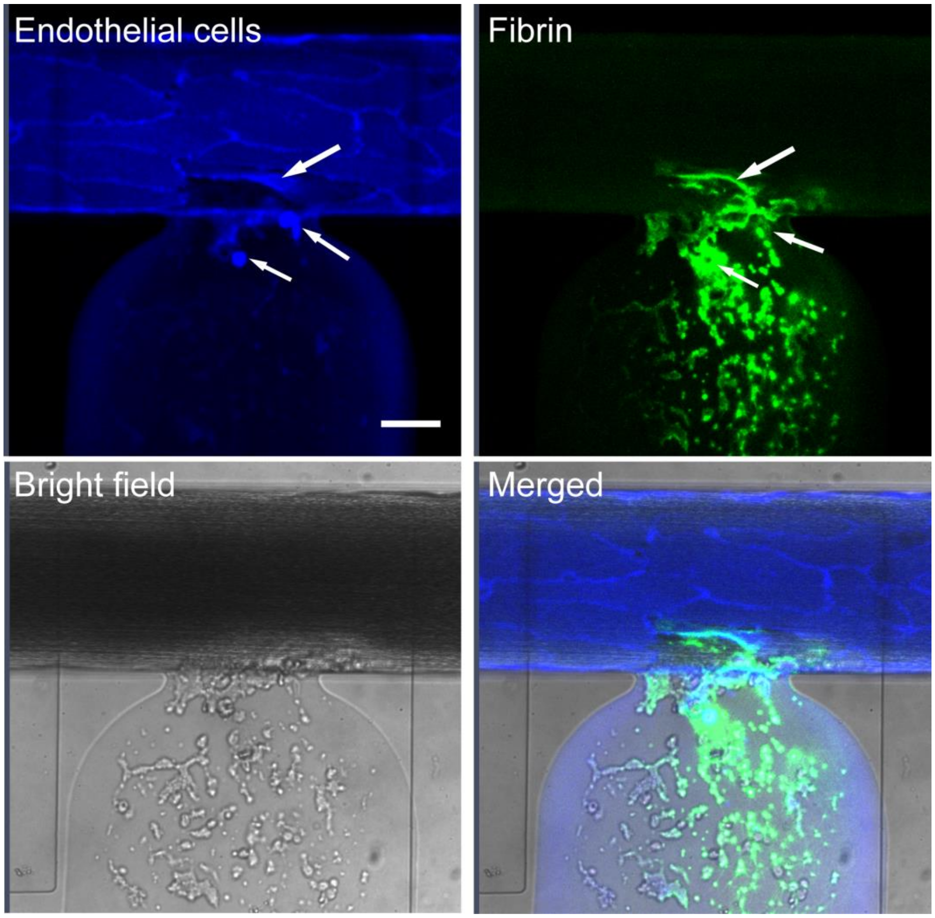

Supplementary Figure 5. Damaged endothelial cells act as a prothrombotic surface for hemostasis. When mechanical injury was introduced, fibrin accumulation (detected via a fibrin specific antibody) was observed at the damaged edge of endothelial cells (large arrow) adjacent to the exposed area and also the detached areas of the endothelial cell membrane (smaller arrows). The image shows the time point 10 min. Scale bar = 50 µm.

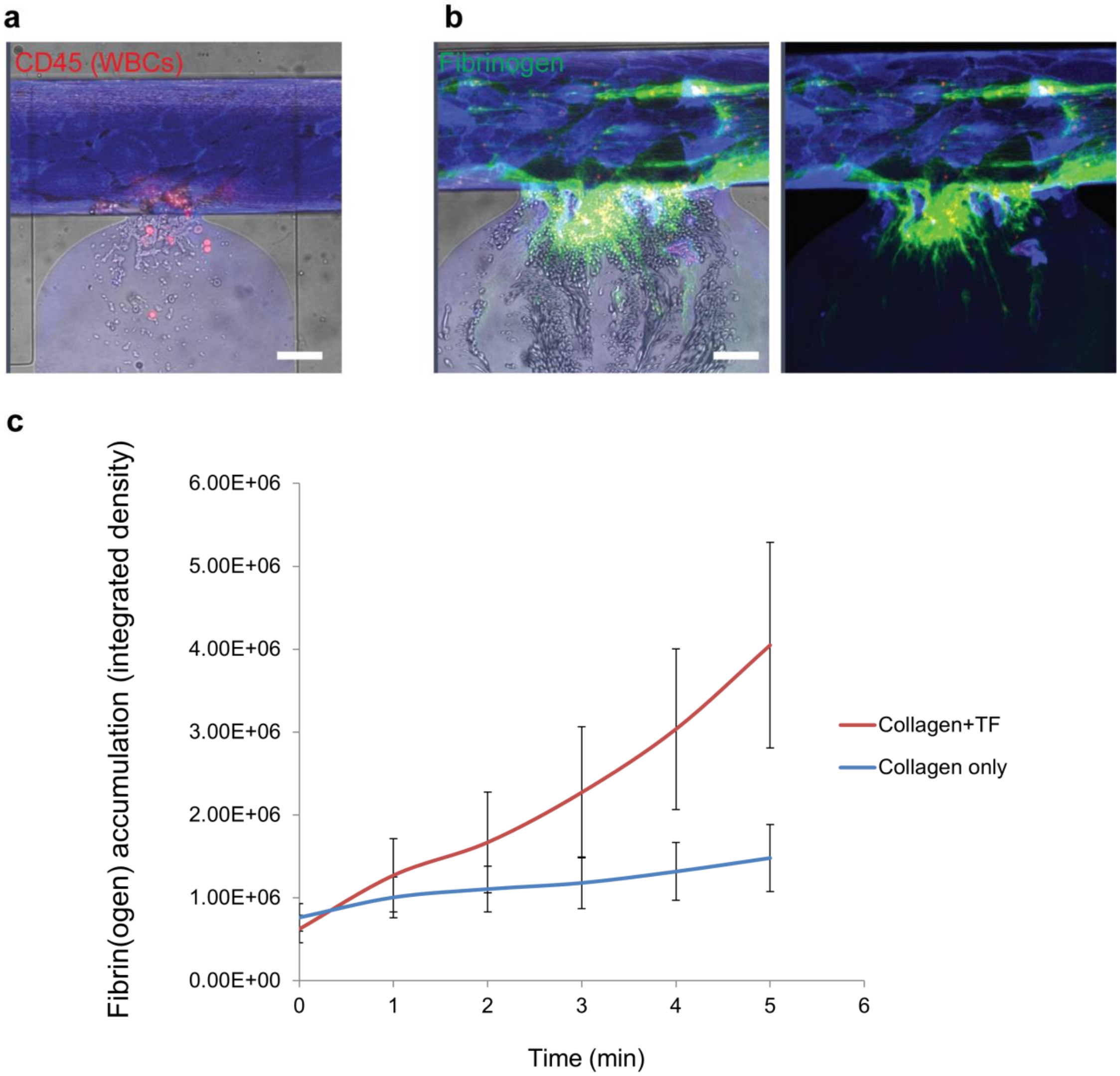

Supplementary Figure 6. Possible involvement of tissue factor in initial thrombin and clot formation. (a) We observed the presence of CD45 positive cells (i.e. white blood cells, red) within the hemostatic plug. (b) When the vascular channel was coated with tissue factor (TF) along with collagen, we observed more fibrin(ogen) (green) accumulated at the wound site at earlier time points. (c) There are significant differences in the amount of fibrin(ogen) accumulation at 5 min (student t-test, $p=0.042$, $n=5$ for each condition, the error bars show standard errors). Scale bars = 50 µm.

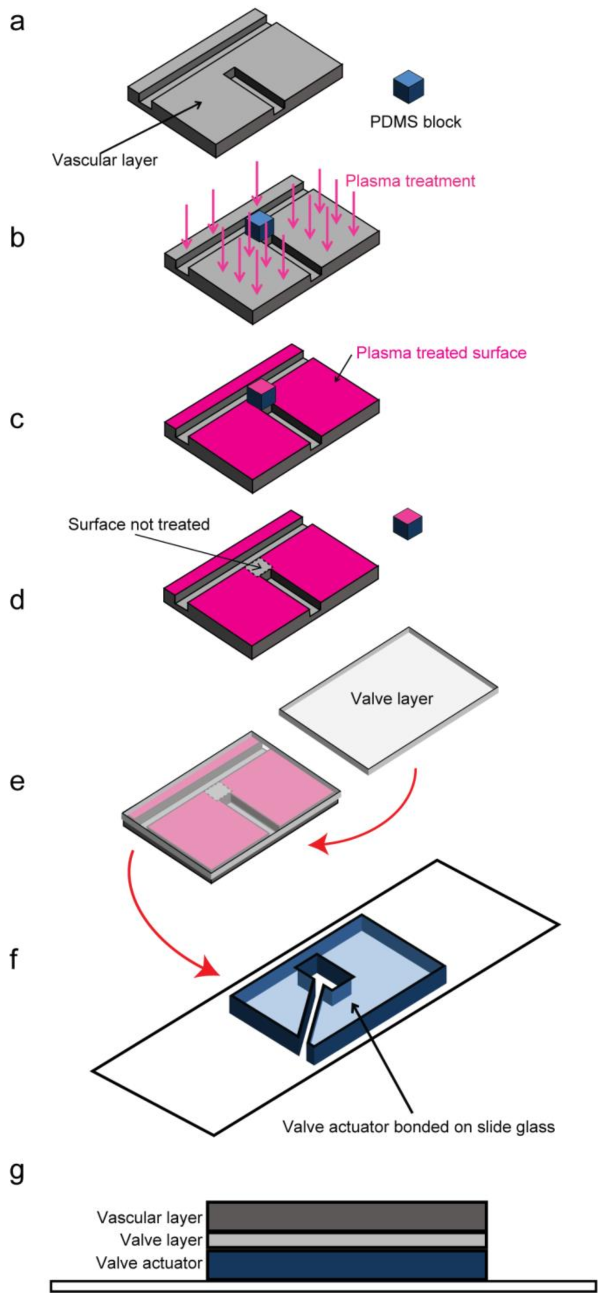

Supplementary Figure 7. The construction of our mechanical injury bleeding model with pneumatic valve. We created a partially bonded microfluidic device with three layers of PDMS. A portion of the vascular layer surface was blocked by a small PDMS block (a) and then oxygen plasma treated (b, c). When the PDMS block was removed, an untreated surface was exposed (d). The plasma-treated vascular layer was attached to the thin PDMS valve layer, bonding only where surface was treated, enabling the untreated region of the valve layer to remain movable (e). The bonded vascular and valve layer was bonded on top of the valve actuator which was pre-bonded to a slide glass (f, g).
