# Supplementary material for: A microengineered vascularized bleeding model that integrates the principal components of hemostasis
Source: Nat Commun. 2018 Feb 6;9:509. doi: 10.1038/s41467-018-02990-x (PMC5802762; doi:10.1038/s41467-018-02990-x)
Supplement: Supplementary file 3 — Description of Additional Supplementary Files [file 41467_2018_2990_MOESM3_ESM.pdf]

Description of Additional Supplementary Files

File Name: Supplementary Movie 1
Description: **Endothelialized microfluidic bleeding device after mechanical injury and subsequent hemostasis**. Blood from a healthy donor is stained with a fluorescently tagged platelet marker (anti-CD41, in red) and contained fluorescently tagged fibrinogen (in green) at 60 μg/ml was perfused into the microdevice where endothelial cells (in blue) were cultured to confluence and mechanical injury was introduced. Time lapse images were obtained via confocal microscopy (6 frames/min) and sped up to and converted to 24 fps Movie. We observed the bleeding via red blood cells flowing through and traversing the wound. We also observed platelet and fibrin(ogen) accumulation over the time course and eventually hemostasis was achieved.

File Name: Supplementary Movie 2
Description: **Intensity map of platelet accumulation.** Blood from healthy donors were treated either with a vehicle control or eptifibatide (10 μg/ml) and were stained with a fluorescent platelet marker (anti-CD41) perfused into the device where endothelial cells were cultured to confluence and mechanical injury was introduced. Time lapse images of fluorescent intensity of the platelets were recorded (6 frames/min) and plotted as an intensity map by MATLAB and sped up to and converted to 24 fps Movie. Here we observed that platelets accumulate, aggregate, and contract to in high density (i.e. dark red) in the control but not in the eptifibatide-treated blood.

File Name: Supplementary Movie 3
Description: **Platelet contraction, control.** Blood from a healthy donor containing a fluorescently tagged platelet marker (anti-CD41, in red) and a 1-2 % subpopulation of platelets (stained with calcein, green) was perfused into the device where endothelial cells were cultured to confluence and mechanical injury was introduced. Time lapse images were obtained via confocal microscopy (6 frames/min) and sped up to and converted to 24 fps Movie. We visually observed that the subpopulation of platelets (indicated with arrows) displaced proximally and against the direction of flow in the vascular channel during the bleeding, thus clot contraction occurs.

File Name: Supplementary Movie 4
Description: **Platelet contraction, eptifibatide.** Blood from a healthy donor treated with eptifibatide (10 μg/ml) containing a fluorescently tagged platelet marker (anti-CD41, in red) and a 1-2 % subpopulation of platelets (stained with calcein, green) was perfused into the device where endothelial cells were cultured to confluence and mechanical injury was introduced. Time lapse images were obtained via confocal microscope (6 frames/min) and sped up to and converted to 24 fps Movie. We observed that the subpopulation of platelets do not displace from the position where they originally integrated into the nascent clot, thus clot contraction was significantly attenuated.

File Name: Supplementary Movie 5
Description: **PSer exposure, fluorescence only.** Blood from a healthy donor containing fluorescently tagged annexin V (red) and a fluorescently tagged platelet marker (anti-CD41, in green) was perfused into the device where endothelial cells (in blue) were cultured to confluence and mechanical injury was introduced. Im mediately after the injury, annexin V binding was observed on the areas with endothelial cells where mechanical injury was induced. We observed platelet adhesion and accumulation on the damaged endothelial cell area but did not show significant annexin V binding during the experiment. Time lapse images were obtained via confocal microscope (6 frames/min) and sped up to and converted to 24 fps Movie.

File Name: Supplementary Movie 6
Description: **PSer exposure, separate channels.** Blood from a healthy donor containing fluorescently tagged annexin V (red) and a fluorescently tagged platelet marker (anti-CD41, in green) was perfused into the device where endothelial cells (in blue) were cultured to confluence and mechanical injury was introduced. Im mediately after the injury, annexin V binding was observed on the areas of endothelial cells where mechanical injury was induced. We observed platelet adhesion and accumulation on the damaged endothelial cell area but did not show significant annexin V binding during the experiment. Time lapse images were obtained via confocal microscope (6 frames/min) and sped up to and converted to 24 fps Movie. In this Movie, each color channel and phase contrast image are separated.

File Name: Supplementary Movie 7
Description: **Neutrophil crawling in the extravascular wound space.** Blood from a healthy donor containing a fluorescently tagged neutrophil marker (anti-CD15, in green) was perfused into the device where endothelial cells were cultured to confluence and mechanical injury was introduced. We observed neutrophils crawling, especially in the extravascular wound channel. Time lapse images were obtained via confocal microscopy (6 frames/min) and sped up to and converted to 24 fps Movie.

File Name: Supplementary Movie 8
Description: **mAb 2-76 treated blood**. Blood from a healthy donor which was treated with vWF inhibitory antibody mAb2-76, stained with a fluorescently tagged platelet marker (anti-CD41, in red), and containing fluorescently tagged fibrinogen (in green) was perfused into the device where endothelial cells (in blue) were cultured to confluence and mechanical injury was introduced. Time lapse images were obtained via confocal microscopy (6 frames/min) and sped up to and converted to 24 fps Movie. We observed repeated re-bleeding over the time course and hemostasis was not achieved during the course of the recording time.

File Name: Supplementary Movie 9
Description: **Hemophilia A patient blood.** Blood from a hemophilia A patient stained with a fluorescently tagged platelet marker (anti-CD41, in red) and containing fluorescently tagged fibrinogen (in green) was perfused into the device where endothelial cells (in blue) were cultured to confluence and mechanical injury was introduced. Time lapse images were obtained via confocal microscopy (6 frames/min) and sped up to and converted to 24 fps Movie. We observed platelet adhesion but little fibrin(ogen) accumulation at the site of the injury. Thus, hemostasis was not achieved even when the recording time was significantly extended far beyond the 20 min limit typical for our system when using healthy control blood.
